# PhenoPlasm: a database of disruption phenotypes for malaria parasite genes

**DOI:** 10.12688/wellcomeopenres.11896.2

**Published:** 2017-07-24

**Authors:** Theo Sanderson, Julian C. Rayner

**Affiliations:** 1 Malaria Programme, Wellcome Trust Sanger Institute, Wellcome Genome Campus, Hinxton, Cambridge, UK

**Keywords:** Malaria, Plasmodium, phenotype, genetic, database, knock-out

## Abstract

Two decades after the first *Plasmodium *transfection, attempts have been made to disrupt more than 3,151 genes in malaria parasites, across five *Plasmodium *species. While results from rodent malaria transfections have been curated and systematised, empowering large-scale analysis, phenotypic data from human malaria parasite transfections currently exists as individual reports scattered across a the literature. To facilitate systematic analysis of published experimental genetic data across *Plasmodium* species, we have built PhenoPlasm ( http://www.phenoplasm.org), a database of phenotypes generated by transfection experiments in all *Plasmodium* parasites. The site provides a simple interface linking citation-backed *Plasmodium *reverse-genetic phenotypes to gene IDs. The database has been populated with phenotypic data on 367 *P. falciparum *genes, curated from 176 individual publications, as well as existing data on rodent *Plasmodium *species from RMgmDB and PlasmoGEM. This is the first time that all available data on *P. falciparum *transfection experiments has been brought together in a single place. These data are presented using ortholog mapping to allow a researcher interested in a gene in one species to see results across other *Plasmodium *species. The collaborative nature of the database enables any researcher to add new phenotypes as they are discovered. As an example of database utility, we use the currently available datasets to identify RAP (RNA-binding domain abundant in Apicomplexa)-domain containing proteins as crucial to parasite survival.

## Introduction

The increasing use of experimental genetics in *Plasmodium spp.* has provided numerous insights into the biology of the malaria parasite ( de Koning-Ward *et al.*, 2015). Nevertheless, to date such results for *P. falciparum* transfection experiments are scattered across a range of journals, with no unified or queryable interface. This means that a researcher whose experiment or analysis identifies a set of genes of interest must devote considerable time to reviewing all available literature if they are to understand what is already known about these genes from previous knockout or other genetic manipulation experiments. To facilitate rapid functional profiling using already established phenotypes, we set out to build a database to contain this information.

There were three key functional requirements for such a database:

### Systematic, and synergistic with existing resources

To allow for automated bioinformatic analyses, it is crucial that the database have a defined, machine-comprehensible, schema for recording phenotypes. It is also important that this schema is compatible with existing resources. The rodent malaria genetically modified parasite database (RMgmDB, http://pberghei.eu; Khan *et al.*, 2013) provides a powerful curated resource for the rodent *Plasmodium* species, and contains curated data on disruption attempts for over 500 genes from individual studies, making it the largest manually curated database for *Plasmodium* experimental genetic data. However, this database does not contain any data for human-infecting *Plasmodium* species. While some human *Plasmodium* parasite genes lack rodent orthologs, nearly 75% have such orthologs, and integrating human and rodent *Plasmodium* phenotypes is likely to be highly informative. To allow such integration, any new database schema must be compatible with that of RMgmDB, which is broken into 6 different stages at which phenotypes can occur (asexual, gametocyte/gamete, fertilization & ookinete, oocyst, sporozoite and liver). RMgmDB also distinguishes cases in which a modification is not successful, which provide some implication of a possible role in asexual growth; we decided to call this quality *mutant viability*, though of course failure to obtain a mutant might also result from a technical failure. The database must also import phenotypes from the largest source of blood stage *Plasmodium* phenotyping data available to date, PlasmoGEM barcode-sequencing experiments ( Bushell *et al.*, 2017).

### Orthology-based retrieval

The use of model systems has strongly facilitated attempts to understand human malaria parasites ( Zuzarte-Luis *et al.*, 2014). These systems are valuable both because rodent malaria parasites are less technically challenging to genetically modify, and because their *in vivo* nature has allowed the study of some aspects of parasite biology in a more physiological setting than provided by *in vitro* culture. Critically, rodent models allow the recapitulation of the complete lifecycle with few technical hurdles, and therefore the vast majority of known non-blood-stage phenotypes come from rodent *Plasmodium* experiments. Nevertheless, rodent parasites do not contain orthologs of every *P. falciparum* gene, so these studies alone cannot provide a complete view of the parasite genetics causing human disease. Even where orthologs exist, phenotypes may not always be conserved, although available comparative data does suggest a high level of conservation ( Bushell *et al.*, 2017).

We felt that an optimal approach would allow a researcher to search for gene IDs from any species but at a glance to see both results in this organism and for orthologous genes in other *Plasmodium* species. The database should also contain records for emerging new genetic models, such as *P. knowlesi* ( Kocken *et al.*, 2002; Moon *et al.*, 2013), as well as *P. vivax* which, though currently genetically intractable, is of key medical importance, and where gene functions may be interpreted through orthology.

### Community contribution

The role for “crowd-sourcing” in biological databases is contentious ( Good & Su, 2013; Karp, 2016). It is clear that community contributions cannot wholly replace curation for these types of datasets, but on the other hand manual curation is not easy to support through application to funding agencies, and suffers the problem of scale - a single person is unlikely to identify every single phenotype in the *Plasmodium* literature. An example of successful community contribution in parasite genetics comes from the EuPathDB databases (which include PlasmoDB, Aurrecoechea *et al.*, 2009). These have had thousands of user comments, many of which are now incorporated into annotations. We felt it important to provide a mechanism whereby a motivated researcher can add any missing phenotypes to the database. This requires the creation of an intuitive interface and easy interface for the entry of data, and means that the primary data source must be publications, with each phenotype backed up with a PubMed ID.

## Methods

### Database schema

The database comprises 4 main data tables. Phenotype data is stored in a “Phenotypes” table, containing the stage at which the phenotype is described, the phenotype itself (selected from a growing and defined taxonomy), a referenceable citation, details of the genetic system used to obtain the phenotype and any additional notes. Here, the gene itself is a reference to the “Genes” table, containing gene name and product data imported from PlasmoDB ( Aurrecoechea *et al.*, 2009). Genes are linked to previous aliases by an “Aliases” table. They are also linked to one another by the “Orthology” table, which contains links between genes in which both OrthoMCL group and synteny is conserved.

### Display

**Figure 1.**
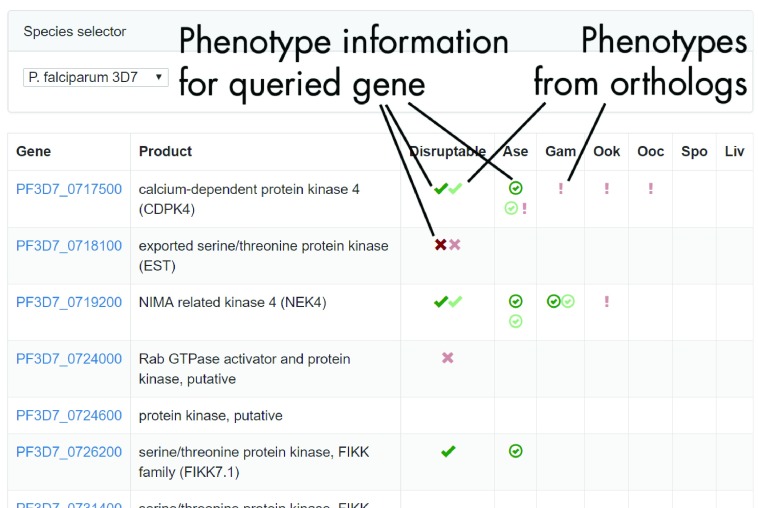
The results of a search for ‘kinase’ genes, showing phenotype data, both from *P. falciparum* experiments and those in rodent models across multiple lifecycle stages. Green ticks indicate mutant viability, and circled green ticks indicate wild-type phenotype. Red crosses indicate failure to disrupt the gene, and red exclamation marks indicate a phenotype different from wildtype. The icons are either shown in full opacity (indicating they apply to the gene in the species queried) or semi-transparent (indicating they refer to orthologous genes in other species).

**Figure 2.**
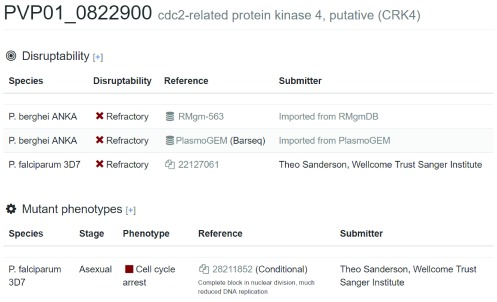
The phenotype page for the *P. vivax* CRK4 gene. Though no experimental data is available directly from *P. vivax*, published results are shown from *P. berghei* and *P. falciparum*, with references to the original datasets from which likely data in *P. vivax* could be inferred. This gene is essential and has therefore been refractory to all attempts to disrupt it by classical reverse genetics, but a conditional system has also been recently applied in *P. falciparum*, allowing a more detailed phenotype to be assigned to the gene from our taxonomy.

The database can be queried either for a set of genes ( Figure 1) or a single gene ( Figure 2). The former provides a table with one line per gene, while the latter provides referencing for each claim and displays any additional notes. The search box on each page can be flexibly queried with a gene ID, symbol or description; but there is also an advanced search facility which allows the retrieval of, for example, only phenotypes backed up by evidence from conditional systems.

### Literature review

A scan was made of the *Plasmodium* literature using Google Scholar (which provides full-text search for a large proportion of publications) to identify reported attempts at *P. falciparum* gene disruption for curation. Terms for which complete literature scans were made included ‘“attempts to disrupt” falciparum’, ‘“gene disruption of” falciparum’ ‘“gene deletion construct” falciparum’ and ‘“gene disruption construct” falciparum’. Numerous additional terms were used, and the first 10 pages of results for each search were manually curated and added to the database. In addition, genes with a suggested role in erythrocyte invasion were systematically curated by searching for all references to any version of their gene IDs, as discussed below.

One challenge when conducting literature searches into *Plasmodium* proteins is the fact that the numerous iterative improvements made to *Plasmodium* genome assemblies mean that a gene could be referred to by any of numerous current or historic gene IDs. To assist with this, the PhenoPlasm page for each gene contains a link to conduct a custom boolean search on Google Scholar, searching article full text for any of the historic gene identifiers which have been historically used to refer to the gene, as provided by PlasmoDB ( Aurrecoechea *et al.*, 2009). Links are also provided to other databases, including searches for pathways and localization images on the Malaria Metabolic Pathways Site.

In addition to this curation, scripts were developed to regularly import data from RMgmDB and PlasmoGEM and transform it to the PhenoPlasm schema.

### Enrichment analysis

Genome-scale phenotyping data provides opportunities to integrate diverse genome-wide data sets and investigate how they relate to gene functionality. The PlasmoGEM dataset, which currently contains data for >50% of *P. berghei* genes, has been used to identify essential metabolic pathways and investigate the relationship between transcriptomics, evolution and phenotype ( Bushell *et al.*, 2017).

To supplement these analyses, and further illustrate the utility of genome-scale phenotype data, we sought to identify protein domains whose presence in a gene was predictive of essentiality or dispensability. We downloaded from PhenoPlasm the phenotypes relating to all *P. falciparum* genes, both directly assayed and inferred from orthologs, and added annotation information for InterPro signatures ( Aurrecoechea *et al.*, 2009). We then used hypergeometric testing to identify signatures with members significantly enriched in essential or dispensable genes ( Supplementary File S1).

## Results

### The extent of data now available on PhenoPlasm

**Figure 3.**
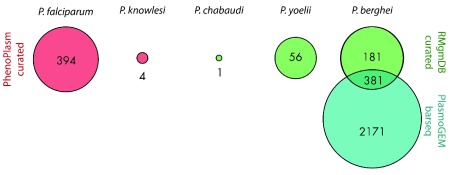
The number of genes with phenotyping data available in PhenoPlasm for each *Plasmodium* species, and the source of these annotations.

At the time of manuscript preparation, some form of phenotyping information is available for 3,188 genes ( Figure 3). Of these, 2,790 are from rodent malaria parasites, and so represent data imported from RMgmDB and PlasmoGEM. The remaining 398 are human parasite genes with phenotypes systematised by our curation, and brought together in a searchable format for the first time. For posterity, the complete data has been additionally deposited on Figshare ( https://doi.org/10.6084/m9.figshare.5114017), and will be updated at least yearly.

There are 7,274 total phenotype datapoints (i.e. data for one life-stage, for one gene knock-out, in one study). The majority of the non-blood stage phenotype datapoints are from the rodent parasites, since relatively few *P. falciparum* genes have phenotyping data reported beyond the blood stage.

Given that some genes have been phenotyped in multiple *Plasmodium* species, the number of ortholog groups covered in at least one species is 2,778. This represents 60% of the core *Plasmodium* genome.

**Figure 4.**
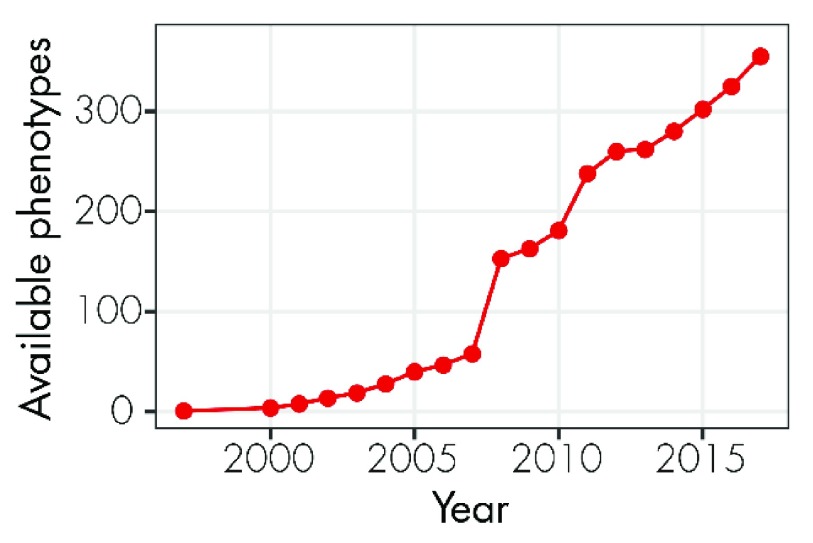
A timeline of PubMed dates associated with publications reporting knock-out phenotypes for *P. falciparum* genes, from the first gene disruption in 1997 to mid-2017. The values shown are cumulative from all previous years. Around 25 genes per year have had disruption attempts reported since the year 2000. The spikes that occur in 2008 and 2011 largely represent two individual publications systematically knocking out exported genes ( Maier *et al.*, 2008) and kinases ( Solyakov *et al.*, 2011) respectively.

Since every phenotype in PhenoPlasm for the human malaria parasites is linked to a PubMed ID or other citation, we were able to informatically extract the dates associated with these publications and plot how the number of genes phenotyped in *P. falciparum* has increased over time ( Figure 4). While this analysis has limitations (a portion of citations are review papers), it does reveal that progress remains slow in human-infecting parasites, and with no major acceleration apparent in the last decade. This illustrates the current importance of rodent models, and raises the question of the technologies needed to create a step-change in the rate of phenotype discovery for human malaria species.

### Applying the data to investigate genome-wide relationships between protein structure and knock-out phenotype reveals the importance of RAP-domain containing proteins

The InterPro signatures most enriched in genes producing viable mutants included the 6-cysteine domain (12/12 viable, p=7.46E-05), the MSP7 C-terminal domain (8/8 viable, p=0.0018) and the MFS transporter superfamily (21/24 viable, p=2.19E-05). These data confirm previous inferences, but does so in a systematic way for the first time ( Supplementary Table S1).

The InterPro signatures enriched in apparently essential genes include expected results, such as the OB-fold nucleic acid domain and ribosomal protein S5 domain-2 type folds, but also identify the RAP (RNA-binding domain abundant in Apicomplexa) domain as functionally highly significant ( Table 1, Supplementary Table S1). The RAP domain was named for its dramatic expansion in the Apicomplexa, as compared to other parts of the tree of life ( Lee & Hong, 2004). Every one of the ten genes containing this domain for which knockouts have been attempted to date appears to be essential (there are 18–19 in most *Plasmodium* genomes). As a result of this analysis, we looked at the other apicomplexan taxon for which genome-scale data is available and found that all eleven proteins containing this domain in *Toxoplasma gondii* also have suggestions of essentiality in CRISPR-screening data ( Sidik *et al.*, 2016). This functional data, combined with recent experimental observations of these proteins binding mRNA ( Bunnik *et al.*, 2016), suggest these proteins as prominent candidates for future studies which may uncover a new realm of Apicomplexa-specific biology.

**Table 1.** InterPro signatures most enriched in essential genes.

InterPro Family	Description	Viable mutant	Essential gene	Proportion of genes essential	p-value
**IPR012340**	Nucleic acid-binding, OB-fold	3	24	89%	0.0002
**IPR020568**	Ribosomal protein S5 domain 2-type fold	2	18	90%	0.0008
**IPR013584**	RAP domain	0	10	100%	0.0023
**IPR006073**	GTP binding domain	0	9	100%	0.0043
**IPR005225**	Small GTP-binding protein domain	4	19	83%	0.0049

## Discussion

A rapid growth in available genetic tools, coupled with decreasing costs of gene synthesis and sequencing, mean that *Plasmodium* experimental genetics is reaching the genome-level scale for the first time. Phenotypic data from these studies has the potential to shed light on the importance of the novel genes found in these early-branching eukaryotes. The development of large scale genetic modification programmes in *Plasmodium* species ( Bronner *et al.*, 2016; Bushell *et al.*, 2017; Gomes *et al.*, 2015) is now shedding light on a large portion of the genome’s functional importance in the asexual blood stage.

Nevertheless, no single approach is likely to reach saturation for some time, and exploring the complete parasite lifecycle in any system is likely to take even longer. In addition, the lack of a non-homologous end-joining pathway in *Plasmodium* parasites prevents the use of the conventional CRISPR-Cas9 screens, which have revolutionized genetics in other organisms ( Sidik *et al.*, 2016). For these reasons, a complete view of the reverse-genetic landscape for a gene or pathway will require bringing together multiple datasets with the individual gene-by-gene studies that have characterized decades of research.

Until now, retrieving phenotyping data for a set of 80 genes in *Plasmodium* might have involved perhaps a day of work, requiring a separate literature search for each gene’s *P. falciparum* ID, and all of its historic identifiers. To be comprehensive, the set of genes would additionally have to be transformed by orthology into each of the five available Plasmodium genetic systems, with further searches conducted. With the development of PhenoPlasm, all this data is available in a single batch search.

We hope that this database will assist in the prioritization of future large-scale studies, eliminating duplication of existing efforts, and allowing a focus on the portion of the genome which remains wholly unexplored by previous reverse genetic approaches. The availability of systematized data should allow the *Plasmodium* phenome to be bioinformatically queried in the same sort of routine way that transcriptomic data is used today to understand gene function. The application we present here, identifying RAP domain proteins as crucial for parasite survival, is just a hint of the wealth of information that well-organized phenotypic data can reveal at scale.

## Data availability

The data referenced by this article are under copyright with the following copyright statement: Copyright: © 2017 Sanderson T and Rayner JC

The database is accessible at http://phenoplasm.org/. Facilities are provided for download of batch searches in CSV form, and of the entire dataset. In addition, snapshots are available from Figshare ( https://doi.org/10.6084/m9.figshare.5114017) which will be updated yearly.

## Supplementary material

**Supplementary Table S1: Full table of InterPro signatures, and the number of viable and non-viable mutants for proteins containing them, with p-values indicating statistical enrichment.**

Click here to access the data

**Supplementary File S1: Reproducible analysis.** The analytical methods used to calculate the results presented here are preserved in an R-Markdown document to allow full reproduction of the results.

Click here to access the data
