# Supplementary material for: PhenoPlasm: a database of disruption phenotypes for malaria parasite genes
Source: Wellcome Open Res. 2017 Jul 24;2:45. Originally published 2017 Jun 21. [Version 2] doi: 10.12688/wellcomeopenres.11896.2 (PMC5500895; doi:10.12688/wellcomeopenres.11896.2)
Supplement: Peer review history

# Peer review: Referee response for version 1

**DOI:** 10.21956/wellcomeopenres.12855.r23827

**Published:** 2017-07-03

**Authors:** Omar S. Harb

**Affiliations:** 1 Institute for Biomedical Informatics, Perelman School of Medicine, Department of Biology, University of Pennsylvania, Philadelphia, PA, USA

This is a nice paper describing the PhenoPlasm database. This database is unique in that in collects information about *Plasmodium falciparum* genetic modifications in one place and enables users to search the database using both systematic gene IDs and text search. Overall I believe this paper describes a very useful resource and will help scientists more easily access data about various types of mutants in *Plasmodium*.  I have a number of minor suggestions and comments that should make this resource even more useful:

  

1. In this sentence “Nevertheless, rodent parasites do not contain orthologs of every *P. falciparum *gene, so these studies alone cannot provide a complete view of the parasite genetics **underlying causing **human disease.”, The words underlying causing right after each other don’t make sense. I think one of these words should be dropped.
2. Community contribution section – it would be useful to highlight the success of the community user comment system that has been in place at EuPathDB databases for over 10 years.  Thousands of crowd-sourced user comments on genes have been entered and many of these have been used to guide curation teams to improve and edit annotation.  This would serve as a nice example of how crowd sourcing works even with scientists!
3. “Genes are linked to previous aliases by an “Aliases” table, and to each other by orthology by the “Orthology” table, which contains links where both OrthoMCL group and synteny is conserved.” This sentence sounds a bit awkward – authors should think about rephrasing.
4. Once a search is run, a table of disrupted genes is reported.  It would be useful if the number of rows (or genes) in the table is reported at the top of the page. 
5. Results table: it would be nice if the header of the table is sticky (or locked) so when a user scrolls down they can still see the column names. I found myself having to keep scrolling up and down.
6. Once you run a search there the table contains various symbols eg. Check marks and exclamation marks. These also differ in color intensity/opacity. It wasn’t clear to me what these mean and I could not find a description of what they mean on the site. Maybe I missed it? Regardless it would be good if those can be provided on mouse over or on the site of the table as a legend. Perhaps taking the figure legend from figure 1 in the paper and making available to all table results on the database.
7. How robust was the google scholar search to identify gene id aliases? Both GeneDB and PlasmoDB keep a list of aliases as well. Did you use these? What about genbank or UniProt accession numbers, have those been included as aliases?
8. In the results section the number or genes reported with information in PhenoPlasm differs slightly than what it on the website. I am assuming this is due to ongoing curation which is great! It would be good to state something like “At the time of manuscript preparation…”
9. “For posterity, the complete data has been additionally deposited on Figshare ( https://doi. org/10.6084/m9. gshare.5114017), and will be regularly updated.” Can you define what regularly updated means? Once a month, once a year? How will this be done? Is there a mechanism in place to do this? How important is this anyway?
10. Data presented in Figure 4 is cute but not sure how much it adds to the paper. I am not against it, I just don’t think it adds anything useful.
11. Literature review using google scholar: I think it is important to provide a full list of terms used as a supplemental table. Also, it might even be better to provide a link to the advanced google scholar search so readers can try them out for themselves.
12. Manual curation: Needs more description as to how this was conducted. Was it only based on google scholar or did it also involved reading of papers. How many individuals were involved in reading papers and was there any attempt to quality control the results?
13. You should be able to extract additional mutant data from user comments in PlasmoDB.  I just did a few quick searches showing that there are some in PlasmoDB not in PhenoPlasm but perhaps this can be a community curation/contribution: http://plasmodb.org/plasmo/im.do?s=94dda7211e47d33c

I have read this submission. I believe that I have an appropriate level of expertise to confirm that it is of an acceptable scientific standard.

# Response

**Published:** 2017-07-24

**Authors:** Theo Sanderson

**Affiliations:** Wellcome Trust Sanger Institute, UK

We are very grateful for this rapid review and the helpful suggestions you have made. We have responded inline below for clarity.

*This is a nice paper describing the PhenoPlasm database. This database is unique in that in collects information about Plasmodium falciparum genetic modifications in one place and enables users to search the database using both systematic gene IDs and text search. Overall I believe this paper describes a very useful resource and will help scientists more easily access data about various types of mutants in Plasmodium.  I have a number of minor suggestions and comments that should make this resource even more useful:*

*In this sentence “Nevertheless, rodent parasites do not contain orthologs of every P. falciparum gene, so these studies alone cannot provide a complete view of the parasite genetics underlying causing human disease.”, The words underlying causing right after each other don’t make sense. I think one of these words should be dropped.*

Thank you. This has been fixed.

*Community contribution section – it would be useful to highlight the success of the community user comment system that has been in place at EuPathDB databases for over 10 years.  Thousands of crowd-sourced user comments on genes have been entered and many of these have been used to guide curation teams to improve and edit annotation.  This would serve as a nice example of how crowd sourcing works even with scientists!*

This is a great point that we have put into the text. We are very keen on allowing community contribution, which is the reason we provide the facility, and it is great to have an example to draw on of how this can work in practice.

*“Genes are linked to previous aliases by an “Aliases” table, and to each other by orthology by the “Orthology” table, which contains links where both OrthoMCL group and synteny is conserved.” This sentence sounds a bit awkward – authors should think about rephrasing.*

We have now done so.

*Once a search is run, a table of disrupted genes is reported.  It would be useful if the number of rows (or genes) in the table is reported at the top of the page.*

Thank you for this suggestion. This has been added and indeed improves the interface.

*Results table: it would be nice if the header of the table is sticky (or locked) so when a user scrolls down they can still see the column names. I found myself having to keep scrolling up and down.*

This is a helpful point that we do accept. It currently poses technical challenges. As an intermediate solution we have added mouseover tooltip text to the icons which indicates which stage they correspond to as well as what the icon represents. We hope to make the column headers stick at a future date.

*Once you run a search there the table contains various symbols eg. Check marks and exclamation marks. These also differ in color intensity/opacity. It wasn’t clear to me what these mean and I could not find a description of what they mean on the site. Maybe I missed it? Regardless it would be good if those can be provided on mouse over or on the site of the table as a legend. Perhaps taking the figure legend from figure 1 in the paper and making available to all table results on the database.*

This is a great point raised by both reviewers which we have now implemented. There is a page listing these on the site but this was somewhat buried.

*How robust was the google scholar search to identify gene id aliases? Both GeneDB and PlasmoDB keep a list of aliases as well. Did you use these? What about genbank or UniProt accession numbers, have those been included as aliases?*

We have clarified this section. The feature uses the PlasmoDB previous IDs (which are now acknowledged in the text) and provides a simple mechanism to conduct a search for “PF3D7_123456 OR MAL13P.123456 OR ….” to identify references to the gene. GenBank and UniProt accession numbers have not been included at this time. We believe relatively few knock-out studies use such identifiers.

*In the results section the number or genes reported with information in PhenoPlasm differs slightly than what it on the website. I am assuming this is due to ongoing curation which is great! It would be good to state something like “At the time of manuscript preparation…”*

Your explanation is exactly correct, and we have implemented this suggestion. Thank you.

*“For posterity, the complete data has been additionally deposited on Figshare ( https://doi. org/10.6084/m9. gshare.5114017), and will be regularly updated.” Can you define what regularly updated means? Once a month, once a year? How will this be done? Is there a mechanism in place to do this? How important is this anyway?*

We were aware that a common concern upon the creation of databases is that should funding run out the database will become defunct and inaccessible, and this was our attempt to defuse such concerns by ensuring the data would be available in perpetuity regardless. We have clarified that this will take place at least yearly. We do not feel that this a major concern for *our *database, but then we suspect that the creators of now-defunct databases did not either, so this seems a sensible insurance policy. This process is currently manual.

*Data presented in Figure 4 is cute but not sure how much it adds to the paper. I am not against it, I just don’t think it adds anything useful.*

We think this history is (somewhat) interesting, and are keen to make it publically available. We had no clear idea what this graph would like until we plotted it, because systematised data is needed to do so. We do accept it is a very minor application of the database which will only be useful if (for instance) cited in a review paper.

*Literature review using google scholar: I think it is important to provide a full list of terms used as a supplemental table. Also, it might even be better to provide a link to the advanced google scholar search so readers can try them out for themselves.*

As in response to the other reviewers, we do accept these critiques. Unfortunately, we do not have systematic records of all terms used. As we discuss below, and in response to the other reviewers, further searches yield very few additional genes, giving us confidence that our review has at least been thorough.

*Manual curation: Needs more description as to how this was conducted. Was it only based on google scholar or did it also involved reading of papers. How many individuals were involved in reading papers and was there any attempt to quality control the results?*

This curation generally involved reading the papers in question, with rare exceptions where results were very clear from the abstract. All were read by a single person with a handful of exceptions which are the result of community contributions. One way in which results have been quality-controlled (though this was not the primary motivation) has been looking for conflicts between our curated P. falciparum data and PlasmoGEM data (Bushell et al., 2017). These should be enriched for curatorial errors but did not uncover any. Nevertheless there are almost certainly curatorial errors in the database, which we hope will be resolved with the eyes of the community, given that publications are the ultimate source authority.

If you refer to attempts to control for the quality of the source data, in general we did not consider this to be necessary.

*You should be able to extract additional mutant data from user comments in PlasmoDB.  I just did a few quick searches showing that there are some in PlasmoDB not in PhenoPlasm but perhaps this can be a community curation/contribution: http://plasmodb.org/plasmo/im.do?s=94dda7211e47d33c*

This is a great suggestion, and thank you for providing the search strategy. We have worked through this list and added a number of datapoints. Reassuringly these are mostly cases of insertional mutagenesis or spontaneous deletions, which we did not conduct specifically conduct searches for in putting together the database.

# Peer review: Referee response for version 1

**DOI:** 10.21956/wellcomeopenres.12855.r23692

**Published:** 2017-06-29

**Authors:** Tania F. de Koning-Ward, Natalie Counihan

**Affiliations:** 1 School of Medicine, Deakin University, Waurn Ponds, VIC , Australia

The journal article by Sanderson and Rayner presents a new database termed ‘PhenoPlasm’ that provides a combined database of reverse genetics information extracted from the rodent *Plasmodium *databases RMgmDB and PlasmoGEM and manual curation of phenotype data from publications that have described reverse genetic experiments in *Plasmodium falciparum*.

 This new database will provide an excellent resource for malaria researchers as it will enable them to very rapidly assess whether attempts to disrupt/knockdown a particular gene(s) in any of the various species has already been attempted and if so what the resulting phenotypes are, without having to transform data by orthology. We actually recommend that the database link is included in the abstract.

 After visiting the database, we noticed there was some additional and very useful features that are not described in the manuscript and we recommend that they are included. For example, the subcellular localisation data and the maps. This puts so much more at the fingertips of researchers. It is ambiguous as to what ‘maps’ refer to until you click on the link so we do recommend that this is called something else, such as ‘pathways protein mapped to’.

 We also recommend that when a search is undertaken that a key of the symbols is visible on the same page for greater clarity. This is clear in the manuscript but not at all evident when using the database.

 Figure 1: For CDPK, there is a circled pale green tick indicating the knockout has a wildtype phenotype. However, next to this is an exclamation mark, meaning the phenotype is different from wildtype. Isn’t the circled green tick and exclamation mark mutually exclusive?

 Figure 2: The word ‘conditional’ is embedded in the text. We were wondering if it would be clearer to instead have an additional column where it could be stated what experimental approach has been used (ie. whether the mutant phenotype data is derived from a gene knockout or conditional knockout/knockdown, etc). For an example, if you search Pf3D7_0929400, the disruptability table indicates the gene is refractory to deletion in *P. falciparum* but in the mutant phenotypes table, the phenotype is ! Attenuated. This is somewhat confusing as parasites are not attenuated until after knockdown.

 Some of the terms that were used in the Literature review section are indicated. However, it would be good to know what the numerous additional terms used were – for example, terms like ‘knockout’, ‘knockdown’, ‘depletion’, ‘conditional’ may have picked up additional publications. For example, HSP101 has been knocked out in *P. berghei, *hence why the cross is opaque in the search result (although this is not evident without a key on this page) but HSP101 has also been conditionally knocked down in *P. falciparum *(Beck *et al*, 2014 Nature) but this is missing from the database. Similarly, other conditional knockouts are missing from the database (Ito *et al*, eLIFE, 2017). (This is why we ticked 'partly' for sufficient details of methods as it wasn't clear what the terms used for inclusion criteria were.)

 Interpro signature section. In the first paragraph (results, page 5), should this be specifically the MSP7 C-terminal domain rather than any of the other MSPs C-terminal domain (for example, MSP1)?

 In Supp. Table 1, the signatures are from various platforms (eg. SMART, EMBL-EBI Pfam, TIGR, etc) and therefore the same domain may be represented more than once. It may be good to note this in the text. Thus, in some cases, the numbers do not match between search engines. (eg. AAA = 7/20 or 17/33).

 This section also mentions the MFS transporter as 21/24 being viable but in the supplemental table MFS is listed twice with 8/9 (PF07690) and 3/4 (PS50850) being viable. Are we missing something in the way we are searching this Table? Moreover, we also couldn’t see the 6 cysteine domain in Supp Table 1.

 There were a couple of minor formatting issues:

1. Delete ‘a’ on line 6. Ie. across a the literature.
2. The quotation marks for terms used in the literature searches need correction.
3. In the results section, second paragraph, italicise *P. falciparum*. Also on Page 6, second paragraph.

We have read this submission. We believe that we have an appropriate level of expertise to confirm that it is of an acceptable scientific standard.

# Response

**Published:** 2017-07-21

**Authors:** Theo Sanderson

**Affiliations:** Wellcome Trust Sanger Institute, UK

Thank you very much for this rapid and highly constructive review which has significantly improved the database. We have been working to address the points you have raised and have responded inline below.

*The journal article by Sanderson and Rayner presents a new database termed ‘PhenoPlasm’ that provides a combined database of reverse genetics information extracted from the rodent Plasmodium databases RMgmDB and PlasmoGEM and manual curation of phenotype data from publications that have described reverse genetic experiments in Plasmodium falciparum.*

*This new database will provide an excellent resource for malaria researchers as it will enable them to very rapidly assess whether attempts to disrupt/knockdown a particular gene(s) in any of the various species has already been attempted and if so what the resulting phenotypes are, without having to transform data by orthology. We actually recommend that the database link is included in the abstract.*

 The database link is indeed included in the abstract.

*After visiting the database, we noticed there was some additional and very useful features that are not described in the manuscript and we recommend that they are included. For example, the subcellular localisation data and the maps. This puts so much more at the fingertips of researchers. It is ambiguous as to what ‘maps’ refer to until you click on the link so we do recommend that this is called something else, such as ‘pathways protein mapped to’.*

 We agree that “Maps” is confusing and have amended accordingly. We have also added a sentence in the text pointing out we include links out to these resources from the Malaria Metabolic Pathways database.

*We also recommend that when a search is undertaken that a key of the symbols is visible on the same page for greater clarity. This is clear in the manuscript but not at all evident when using the database.*

 This is an excellent point raised by both reviewers which we have now addressed with an online key.

*Figure 1: For CDPK, there is a circled pale green tick indicating the knockout has a wildtype phenotype. However, next to this is an exclamation mark, meaning the phenotype is different from wildtype. Isn’t the circled green tick and exclamation mark mutually exclusive?*

 This is an important point illustrated by the phenotypes shown for CDPK4. These phenotypes are indeed in contradiction. This is a genuine case of mutually exclusive experimental reports. Our response to such situations is to display both contradictory phenotypes. This should alert the researcher to a controversy. Clicking on the gene in question allows the sources of the two phenotypes to be traced and a judgement made.

 In this case there are two reports on RMgmDB of “No difference” for growth in the asexual bloodstage. However PlasmoGEM barseq data suggests a subtle 14% decrease in growth rate. It is possible that a relatively small attenuation like this might be missed with other methodologies, so this is a relatively minor contradiction. The database will also contain less reconcilable differences. At this stage our aim is to present the interested scientist with all reported phenotypes and allow them to draw their own conclusions.  

*Figure 2: The word ‘conditional’ is embedded in the text. We were wondering if it would be clearer to instead have an additional column where it could be stated what experimental approach has been used (ie. whether the mutant phenotype data is derived from a gene knockout or conditional knockout/knockdown, etc). For an example, if you search Pf3D7_0929400, the disruptability table indicates the gene is refractory to deletion in P. falciparum but in the mutant phenotypes table, the phenotype is ! Attenuated. This is somewhat confusing as parasites are not attenuated until after knockdown.*

 We do understand this concern. In light of this suggestion we tested implementing the suggestion of a column, but ultimately decided it was slightly more confusing simply because of the increased busyness of each phenotype. We will keep this suggestion in mind for the future as we receive more feedback.

*Some of the terms that were used in the Literature review section are indicated. However, it would be good to know what the numerous additional terms used were – for example, terms like ‘knockout’, ‘knockdown’, ‘depletion’, ‘conditional’ may have picked up additional publications. For example, HSP101 has been knocked out in P. berghei, hence why the cross is opaque in the search result (although this is not evident without a key on this page) but HSP101 has also been conditionally knocked down in P. falciparum (Beck et al, 2014 Nature) but this is missing from the database. Similarly, other conditional knockouts are missing from the database (Ito et al, eLIFE, 2017). (This is why we ticked 'partly' for sufficient details of methods as it wasn't clear what the terms used for inclusion criteria were.)*

 Thank you for pointing out these omissions of specific studies, which we have now rectified. Many of these further search terms were in fact used, but we accept this critique as it would have been useful to have kept an exhaustive list of queries used.  We feel we are now beyond the point of diminishing returns where additional searches have yielded very few new results. For instance the ‘ depletion’ search did not recover any new results in the first 10 pages. There are no doubt further missing phenotypes but we feel these will be best added by engaging with the community.

*Interpro signature section. In the first paragraph (results, page 5), should this be specifically the MSP7 C-terminal domain rather than any of the other MSPs C-terminal domain (for example, MSP1)?*

 Thank you, we have changed this. We were accurately quoting the InterPro family name but we agree that more specificity is helpful in the text here.

*In Supp. Table 1, the signatures are from various platforms (eg. SMART, EMBL-EBI Pfam, TIGR, etc) and therefore the same domain may be represented more than once. It may be good to note this in the text. Thus, in some cases, the numbers do not match between search engines. (eg. AAA = 7/20 or 17/33).*

 Yes - we had similar concern , we have now associated phenotypes not with the signature itself but with the parental InterPro family and re-run the analysis. This does not significantly impact the results scientifically but we agree that it makes it easier to interpret the data by avoiding duplicated rows and associating better descriptive names.

*This section also mentions the MFS transporter as 21/24 being viable but in the supplemental table MFS is listed twice with 8/9 (PF07690) and 3/4 (PS50850) being viable. Are we missing something in the way we are searching this Table? Moreover, we also couldn’t see the 6 cysteine domain in Supp Table 1.*

 The data here was correct, but limited annotation made it confusing to interpret. The signature SSF103473 was 21/24 viable, and does correspond to the MFS InterPro family but the SSF103473 family itself does not have a descriptive name. The same applies to 6-cysteine protein family. Now that we are using InterPro families instead all this confusion should be resolved, since the InterPro family name is listed in the table.

*There were a couple of minor formatting issues:*

1. *Delete ‘a’ on line 6. Ie. across a the literature.*
2. *The quotation marks for terms used in the literature searches need correction.*
3. *In the results section, second paragraph, italicise P. falciparum. Also on Page 6, second paragraph.*

Thank you, these have been fixed.

 ___

 Theo Sanderson
